# Impact of exercise on renal function, oxidative stress, and systemic inflammation among patients with type 2 diabetic nephropathy

**DOI:** 10.4314/ahs.v22i3.30

**Published:** 2022-09

**Authors:** Mohamed H Saiem Aldahr, Shehab M Abd El-Kader

**Affiliations:** 1 Department of Medical Laboratory Technology, Faculty of Applied Medical Sciences, King Abdulaziz University, Jeddah, Saudi Arabia; 2 Department of Physical Therapy, Faculty of Applied Medical Sciences, King Abdulaziz University, Jeddah, Saudi Arabia

**Keywords:** Aerobic Exercise, Diabetic Nephropathy, Inflammatory Cytokines, Oxidative Stress

## Abstract

**Background:**

Diabetic nephropathy (DN) is a prevalent microvascular diabetic complication all over the world.

**Objective:**

This study was designed to measure oxidative stress, systemic inflammation and kidney function response to exercise training in patients with type 2 diabetic (T2DM) nephropathy.

**Material and Methods:**

Eighty obese T2DM patients (50 males and 30 females), their body mass index (BMI) mean was 33.85±3.43 Kg/m^2^ and the mean of diabetes chronicity was 12.53±2.64 year participated in the present study and enrolled two groups; group I: received aerobic exercise training and group II: received no training intervention.

**Results:**

The mean values of creatinine, interleukin-6 (IL-6), tumor necrosis factor-alpha (TNF-α) and malondialdehyde (MDA) were significantly decreased, while the mean values of interleukin-10 (IL-10), glutathione peroxidase (GPx) and glutathione (GSH) were significantly increased in group (A) after the aerobic exercise training, however the results of the control group were not significant. In addition, there were significant differences between both groups at the end of the study (P<0.05).

**Conclusion:**

There is evidence that aerobic exercise training modulated oxidative stress and inflammatory cytokines and improved renal function among patients with diabetic nephropathy.

## Introduction

Diabetic nephropathy (DN) considered as the most serious diabetic complication; while renal replacement is required for the majority of subjects with chronic renal disease among patients with T2DM^1^,^2^, where poor glycemic control^3^ is related to abnormal oxidative stress and systemic inflammation that induce progressive diabetic renal lesion^4^,^5^. Hyperglycemia induces oxidative stress and inflammation^6^. In addition, poor glycemic control induces abnormal level of oxidative stress markers^7^. In the other hand, oxidative stress induce dysfunction of β-cell that lead to insulin resistance development, diabetes and its associated microvascular complications^8^,^9^, so that patients with T2DM are under oxidative stress because of prolonged exposure to hyperglycemia^10^.

Researches proved that hyperglycemia that induced systemic inflammation and oxidative stress which induce DN^11^,^12^. Hyperglycemia in diabetic patients leads to mitochondrial dysfunction, advanced glycation end processes and other factors, and generate the reactive free radicals, then triggers the DNA fragmentation that lead to cell death^13^. However, Navarro et al. found an increase in the gene expression for pro-inflammatory cytokine in patients with DN^14^. Several studies reported that there was a significant elevation in inflammatory cytokines in T2DM with DN and there is an association between their levels and the incidence & the course of renal lesion among diabetics^15^–^17^.

Hyperglycemia also causes oxidative stress, decreases the regeneration of glutathione (GSH) from oxidized GSH and reduces the availability of nicotinamide adenine dinucleotide phosphate^18^,^19^. However, several reports stated that there was reduced level of GSH in diabetes associated with systemic inflammation^20^–^22^. In addition, in β-cell dysfunction may be related to abnormal GSH level induce long-term complications of diabetes^23^. Moreover, low GSH is related to DNA oxidative damage in T2DM^24^. Many studies reported decline in the level of SOD in diabetic tissue and blood^25^,^26^. While, study performed by Lucchesi and colleagues to observe the oxidative balance of diabetic rats reported diminished activity of SOD and other antioxidative enzymes in the liver tissue^27^. In the other hand, several studies reported an increased MDA level in patients with T2DM^28^,^29^. In addition, Baynes and Ramesh et al. reported that lipid peroxidation in diabetes induced many secondary chronic complications including atherosclerosis and neural disorders^30^,^31^.

Physical activity has several health benefits and plays an important role in treatment of chronic disorders. However, regular physical activity improves glucose control, blood lipid profile, insulin sensitivity and endothelial function that help to prevent diabetic complications^32^. Moreover, physical activity may reduce the risk and progression for diabetic nephropathy^33^.

This study was designed to measure oxidative stress, systemic inflammation and kidney function response to exercise training in patients with type 2 diabetic nephropathy.

## Materials and Methods

### Subjects

**Figure 1:**
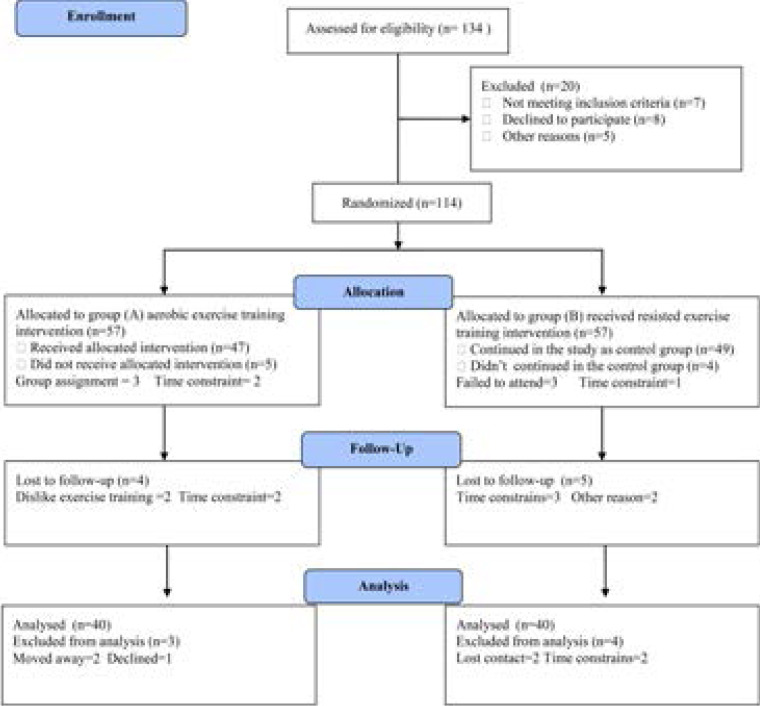
Subjects screening and recruitment CONSORT diagram.

Eighty obese T2DM patients (50 males and 30 females), their body mass index (BMI) mean was 33.85±3.43 Kg/m2and the mean of diabetes chronicity was 12.53±2.64 year participated in the present study and enrolled two groups; group I: received aerobic exercise training and group II: received no training intervention. Exclusion criteria included smokers, kidney insufficiency, congestive heart failure, pregnant female patients, hepatitis and respiratory failure. Clinical evaluations and laboratory analysis were performed by independent assessors who were blinded to group assignment and not involved in the routine treatment of the patients. The CONSORT diagram outlining the details of the screening, run-in and randomization phases of the study and reasons for participant exclusion can be found in figure (1). Informed consent was obtained from all participants. This study was approved by the Scientific Research Ethical Committee, Faculty of Applied Medical Sciences at King University.

### Measurements and procedures

#### A. Measurement of oxidative stress markers and anti-oxidant status

For all participants serum (from 10 ml blood in plain vial) and plasma (from 5 ml blood in EDTA vial) were separated from the sample within 30 min of collection and was stored in pyrogen free polypropylene cryo-tubes at (-80°C) until analysis. Assessment of lipid markers for peroxidation as malondialdehyde (MDA) was determined according to Buege and Aust^34^. However, Anti-oxidant status, glutathione (GSH) that was determined by the method of Beutler and colleagues^35^, in the other hand, glutathione peroxidase (GPx) was measured by the method of Nishikimi and colleagues^36^.

#### B. Measurement of inflammatory cytokines and serum creatinine

Blood samples were drained from the antecubital vein after a 12-hour fasting, the blood samples were centrifuged at + 4 °C (1000 = g for 10 min). Interleukin-6 (IL-6) and Interleukin-10 (IL-10) levels were analyzed by “Immulite 2000” immunassay analyzer (Siemens Healthcare Diagnostics, Deerfield, USA). However, tumor necrosis factor-alpha (TNF-α) was measured by ELISA kits (ELX 50) in addition to ELISA microplate reader (ELX 808; BioTek Instruments, USA). However, serum creatinine was measured with a kit obtained from Stanbio Laboratory (USA).

#### C. Aerobic exercise training program

Patients in group (A) were submitted to a 40 min aerobic session on a treadmill (the initial, 5-minute warm-up phase performed on the treadmill at a low load, each training session lasted 30 minutes and ended with 5-minute recovery and relaxation phase) either walking or running, based on heart rate, until the target heart rate was reached, according to American College of Sport Medicine guidelines. The program began with 10 min of stretching and was conducted using the maximal heart rate index (HRmax) estimated by: 220-age, with exercise intensity was 70–80% of HRmax^34^.

### Statistical analysis

The mean values of the investigated parameters obtained before and after three months in both groups were compared using paired “t” test. Independent “t” test was used for the comparison between the two groups (P<0.05).

## Results

Eighty obese patients with type 2 diabetes mellitus completed the screening evaluation. The baseline characteristics of the participants are shown in table (1). Most participants (60%) were men. Forty participants were assigned group (A) (n = 40; 24 males and 16 females) and group (B) (n =40, 26 males and 14 females). None of the baseline characteristics differed significantly between the two groups is listed in table (1).

**Table (1):** Baseline characteristics of all participants

	Group (A)	Group (B)	Significance
**Age** (year)	48.34 ± 6.91	47.65 ± 7.28	P > 0.05
**Gender** (male/female)	24/16	26/14	P > 0.05
**BMI** (kg/m^2^)	34.15 ± 3.39	33.82 ± 3.47	P > 0.05
**Duration of diabetes** (year)	13.12 ± 2.56	11.94 ± 2.72	P > 0.05
**SBP** (mmHg)	148.53 ± 12.16	145.81 ± 13.44	P > 0.05
**DBP** (mmHg)	92.62 ± 8.75	90.25 ± 7.28	P > 0.05
**HBA1c** (%)	8.52 ± 2.43	8.37 ± 2.21	P > 0.05
**Glucose** (mmol/L)	5.71 ±1.65	5.42 ± 1.48	P > 0.05
**QUICKI**	0.149 ± 0.017	0.158 ± 0.016	P > 0.05
**HOMA-IR**	5.13 ± 1.45	4.71 ± 1.32	P > 0.05

**BMI:** Body Mass Index; **SBP:** Systolic blood pressure; **DBP:** Diastolic blood pressure; **HBA1c:** glycosylated hemoglobin; **QUICKI:** The quantitative insulin-sensitivity check index; **HOMA-IR:** Homeostasis Modl Assessment-Insulin Resistance Index.

The mean values of creatinine, interleukin-6 (IL-6), tumor necrosis factor- alpha (TNF-α) and malondialdehyde (MDA) were significantly decreased, while the mean values of interleukin-10 (IL-10), glutathione peroxidase (GPx) and glutathione (GSH) were significantly increased in group (A) after the aerobic exercise training(table 2), however the results of the control group were not significant (table 3). In addition, there were significant differences between both groups at the end of the study (table 4).

**Table 2:** Mean value and significance of creatinine, MDA, GSH, GPX, TNF-α, IL-6 and IL-10 in group (A) before and after treatment

	Mean + SD		t-value	Significance
	Before	After		
**Creatinine** (µmol/mol)	86.41 ± 7.63	67.92 ± 5.15*	9.24	P <0.05
**MDA** (nM/mL)	0.32 ± 0.07	0.19 ± 0.06*	5.12	P <0.05
**GSH** (nM/mL)	3.54 ± 0.91	4.77 ± 1.23*	6.48	P <0.05
**GPX** (UI/mL)	2.73 ± 0.82	3.95 ± 1.15*	6.26	P <0.05
**TNF-α** (pg/mL)	5.36 ± 1.54	3.18 ± 1.32*	6.17	P <0.05
**IL-6** (pg/mL)	2.85 ± 0.93	1.71 ± 0.67*	5.32	P <0.05
**IL-10** (pg/ml)	6.24 ± 1.61	8.45 ± 1.73*	7.12	P <0.05

MDA: Malondialdehyde; GSH: Glutathione; GPx: Glutathione peroxidase; TNF-α: tumor necrosis factor – alpha; IL-6: Interleukin-6; IL-10: Interleukin-10

* indicates a significant difference between the two groups, P < 0.05.

**Table 3:** Mean value and significance of creatinine, MDA, GSH, GPX, TNF-α, IL-6 and IL-10 in group (B) before and at the end of the study

	Mean + SD		t-value	Significance
	Before	After		
**Creatinine** (µmol/mol)	84.97 ± 6.85	87.26 ± 7.13	1.81	P >0.05
**MDA** (nM/mL)	0.30 ± 0.08	0.33 ± 0.07	0.92	P >0.05
**GSH** (nM/mL)	3.62 ± 0.87	3.56 ± 0.79	1.15	P >0.05
**GPX** (UI/mL)	2.84 ± 0.93	2.67 ± 0.81	1.23	P >0.05
**TNF-α** (pg/mL)	4.88 ± 1.37	5.25 ± 1.39	1.13	P >0.05
**IL-6** (pg/mL)	2.55 ± 0.72	2.86 ± 0.73	0.95	P >0.05
**IL-10** (pg/ml)	6.43 ± 1.54	6.12 ± 1.46	0.92	P >0.05

MDA: Malondialdehyde; GSH: Glutathione; GPx: Glutathione peroxidase; TNF-α: tumor necrosis factor – alpha; IL-6: Interleukin-6; IL-10: Interleukin-10.

**Table 4:** Mean value and significance of creatinine, MDA, GSH, GPX, TNF-α, IL-6 and IL-10 in group (A) and group (B) at the end of the study

	Mean + SD		t-value	Significance
	Group (A)	Group (B)		
**Creatinine** (µmol/mol)	67.92 ± 5.15*	87.26 ± 7.13	8.52	P <0.05
**MDA** (nM/mL)	0.19 ± 0.06*	0.33 ± 0.07	5.37	P <0.05
**GSH** (nM/mL)	4.77 ± 1.23*	3.56 ± 0.79	5.42	P <0.05
**GPX** (UI/mL)	3.95 ± 1.15*	2.67 ± 0.81	5.35	P <0.05
**TNF-α** (pg/mL)	3.18 ± 1.32*	5.25 ± 1.39	5.46	P <0.05
**IL-6** (pg/mL)	1.71 ± 0.67*	2.86 ± 0.73	5.28	P <0.05
**IL-10** (pg/ml)	8.45 ± 1.73*	6.12 ± 1.46	6.33	P <0.05

MDA: Malondialdehyde; GSH: Glutathione; GPx: Glutathione peroxidase; TNF-α: tumor necrosis factor – alpha; IL-6: Interleukin-6; IL-10: Interleukin-10; (*) indicates a significant difference between the two groups, P < 0.05.

## Discussion

Diabetic nephropathy (DN) is a worldwide prevalent medical problem affecting 20–40% of T2DM and characterized with high rate of morbidity and mortality as the DN is a principal etiology of renal failure^35^–^37^. Poor metabolic control, diabetes duration, race, heredity, life style, diet composition, aging, hypertension, systemic inflammation and oxidative stress are the common risk factors of DN^38^,^39^.

Our results demonstrate that aerobic exercise training reduced levels of TNF-α and IL-6, in addition to increased level of IL-10 that indicated reduced systemic inflammation. our results agreed with several studies have shown that aerobic exercise training promotes modulation of inflammatory cytokines^40^–^42^. Several large cohort studies have found a relationship between self-reported physical activity levels and systemic markers of inflammation: higher levels of physical activity are coupled to lower levels of circulating inflammatory markers in elderly individuals^43^–^45^. While, Nicklas et al. showed that regular aerobic exercise training was efficient in lowering IL-6 levels even without weight loss^46^. In addition, Santos and colleagues had twenty-two male, sedentary, healthy, elderly volunteers performed moderate aerobic exercise training for 60 min/day, 3 days/week for 24 week and concluded that 6 months of aerobic exercise training can improve sleep in the elderly via anti-inflammatory effect of aerobic training which modiies cytokine profiles (reduced IL-6 and TNF-α and increased IL-10)^47^. However, Kohut et al. reported that 10-months of aerobic, but not resistance exercise, significantly reduces serum inflammatory mediators in older adults^48^. Moreover, Bote et al. demonstrated that 8-months (2 sessions/week, 60-min/session) of aquatic-based exercise training tempered neutrophil activation (chemotaxis) and decreased systemic levels of IL-8 and noradrenalin compared to controls^49^. Similarly, Ploeger et al. reported that moderate aerobic exercise training has been recommended as an anti-inflammatory therapy^50^. The three possible mechanisms of exercise anti-inflammatory effects include reduction in visceral fat mass^51^; reduction in the circulating numbers of pro-inflammatory monocytes^52^ and an increase in the circulating numbers of regulatory T cells^53^. Moreover, Hong and colleagues show that cardiorespiratory fitness is associated with reduced low grade inflammation which may in part be mediated by enhancing the ability of immune cells to suppress inflammatory responses via adrenergic receptors^54^.

Concerning results of oxidative stress markers, results of our study agreed with other authors who reported that a six-months aerobic exercise was able to decrease lipid peroxidation, as well as to increase GSH and catalase activity in T2DM patients^55^,^56^. A similar study in obese individuals reported attenuation in exercise induced lipid peroxidation following 24 weeks of a moderate intensity resistance training^57^. In addition, Oliveira et al. compared the effects of 12 weeks training with three different types of exercise (aerobic training, strength training and combined training) on T2DM male and female human subjects, demonstrating that the aerobic exercise may help in minimizing oxidative stress and the development of the chronic complications of diabetes^58^. However, Vinetti et al. randomly assigned twenty male subjects with T2DM to an intervention group in a supervised exercise training (SET) consisted of a 12-month supervised aerobic, resistance and flexibility training. They concluded that SET was effective in improving cardiorespiratory fitness, cardiometabolic risk and oxidative stress status in T2DM^59^. While, Farinha et al. completed a 12-week treadmill exercise training, without modifications on dietary pattern in twenty-three women metabolic syndrome who had improved systemic oxidative stress and inflammatory biomarkers^60^. Similarly, Nojima et al. reported that 103 patients with type 2 diabetes mellitus were instructed to exercise at 50% of peak oxygen uptake for more than 30 minutes on at least 3 days per week over a 12 month period, their results proved that aerobic exercise training improved glycemic control and reduced oxidative stress in patients with type 2 diabetes mellitus^61^. Moreover, Gordon et al. reported that 3 months of Hatha yoga exercise and conventional exercise may have therapeutic preventative and protective effects on diabetes mellitus by decreasing oxidative stress and improving antioxidant status^62^. There are 2 mechanisms that underlie the anti-oxidative of aerobic exercise training. The first mechanism is that improvement in glycemic control associated with aerobic exercise training may result in a decrease in oxidative stress. Aerobic exercise training improves insulin sensitivity^63^ and glycemic control^64^. Hyperglycemia can induce oxidative stress via several mechanisms including glucose autoxidation, formation of advanced glycation end products, and activation of the polyol pathway^65^. Chugh et al. reported previously that 6 weeks of glycemic control with sulfonylurea resulted in an improvement of glycemic control and a reduction in serum malondialdehyde, a reliable measure of lipid peroxidation^66^. The other mechanism is that a decrease in oxidative stress caused by aerobic exercise training may lead to an improvement in glycemic control. Aerobic exercise may increase antioxidant activity and reduce oxidative stress. Elosua et al. reported that aerobic exercise training increased the activity of the endogenous antioxidants, glutathione peroxidase, and glutathione reductase and decreased oxidized low-density lipoprotein concentration^67^. There is evidence that oxidative stress is associated with insulin resistance, as Urakawa et al. demonstrated that plasma isoprostane levels were negatively correlated with glucose infusion rates in men^68^. These results therefore indicate that improved insulin sensitivity and glycemic control induced reduction in oxidative stress caused by aerobic exercise training.

Concerning renal function, results of the present study proved that aerobic exercise training improved creatinine in patients with DN, the possible cause for improving renal function following aerobic training may be due modulation of inflammatory cytokines and oxidative. Our results consistent with the studies of Chen et al., Shikano et al. and, Kafle et al. who confirmed the possible role of IL-6 and TNF-α and Gpx in diabetic renal damage progress^69^–^71^. While, Xu et al. conducted a cohort study on 176 patients with chronic kidney disease and 67 healthy controls and reported increased level of CRP, IL-6 and MDA in addition to decreased levels of SOD and GSHPX (glutathione peroxidase) along with inverse relationship between estimated glomerular filtration rate (eGFR) and MDA associated with positive relationship with SOD and GSH-PX among patients with chronic kidney disease (CKD)^72^. Moreover, Aslan et al. reported significant correlations between oxidative stress and microalbuminuria levels in patients with diabetic nephropathy^73^. However, Sreeram et al. reported that among 108 CKD patients, as the renal damage progressed the values of MDA & CRP increased while the values of GPx and SOD decreased^74^. The current study has important strengths and limitations. The major strength is the supervised nature of the study. However, all exercise sessions were supervised. Moreover, the study was randomized; hence, we can extrapolate adherence to the general population. In the other hand, the major limitations is only obese type 2 diabetic patients enrolled in the study, so the value of this study only related to obese patients with type 2 diabetic nephropathy, also small sample size in both groups may limit the possibility of generalization of the findings in the present study. Finally, within the limit of this study, aerobic exercise training is recommended for modulation of oxidative stress and inflammatory cytokines and improved renal function among patients with diabetic nephropathy. Further researches are needed to explore the impact of weight reduction on quality of life and other biochemical parameters among obese patients with type 2 diabetic nephropathy.

## Conclusion

The current study provides evidence that aerobic exercise training modulated oxidative stress and inflammatory cytokines and improved renal function among patients with diabetic nephropathy.
